# Navβ2 knockdown improves cognition in APP/PS1 mice by partially inhibiting seizures and APP amyloid processing

**DOI:** 10.18632/oncotarget.21849

**Published:** 2017-10-16

**Authors:** Tao Hu, Zhangang Xiao, Rui Mao, Bo Chen, Min-Nan Lu, Jun Tong, Rong Mei, Shan-Shan Li, Zhi-Cheng Xiao, Lian-Feng Zhang, Yan-Bin Xiyang

**Affiliations:** ^1^ Institute of Neuroscience, Basic Medical College, Kunming Medical University, Kunming, Yunnan, PR China; ^2^ Department of Laboratory Medicine, The Third People’s Hospital of Yunnan Province, Kunming, Yunnan, PR China; ^3^ Laboratory of Molecular Pharmacology, Department of Pharmacology, School of Pharmacy, Southwest Medical University, Luzhou, Sichuan, PR China; ^4^ Key Laboratory of Medical Electrophysiology, Ministry of Education, School of Pharmacy, Southwest Medical University, Luzhou, Sichuan, PR China; ^5^ School of Stomatology, Kunming Medical University, Kunming, Yunnan, PR China; ^6^ Experiment Center for Medical Science Research, Kunming Medical University, Kunming, Yunnan, PR China; ^7^ Physical Education Department, Kunming Medical University, Kunming, Yunnan, PR China; ^8^ Department of Neurology, The First People’s Hospital of Yunnan Province, Kunming, Yunnan, PR China; ^9^ Basic Medical College, Kunming Medical University, Kunming, Yunnan, PR China; ^10^ Institute of Molecular and Clinical Medicine, Kunming Medical University, Kunming, Yunnan, PR China; ^11^ Monash Immunology and Stem Cell Laboratories (MISCL), Monash University, Clayton, VIC, Australia; ^12^ Key Laboratory of Human Diseases Comparative Medicine, Ministry of Health, Institute of Laboratory Animal Science, Chinese Academy of Medical Sciences(CAMS) & Comparative Medicine Centre, Peking Union Medical College (PUMC), Beijing, China

**Keywords:** voltage-gated sodium channels beta 2, Alzheimer’s disease, APP/PS1 mouse, neuronal activity, cognition, Gerotarget

## Abstract

Voltage-gated sodium channels beta 2 (Navβ2, encoded by SCN2B) is a substrate of β-site amyloid precursor protein cleaving enzyme 1 (BACE1) and regulates cell surface expression of channels in neurons. Previous studies reported enhanced Navβ2 processing by BACE1 in Alzheimer’s disease (AD) model and patients. We investigated whether changes in Navβ2 expression affect neuronal seizure and amyloid precursor protein (APP) processing in an AD mouse model. Our study used eight-month-old APP/presenilin 1 (PS1) mice and transgenic Navβ2 knockdown [by 61% vs. wild type (WT)] APP/PS1 mice (APP/PS1/Navβ2-kd), with age-matched WT and Navβ2 knockdown (Navβ2-kd) mice as controls. We found that Navβ2 knockdown in APP/PS1 mice partially reversed the abnormal Navβ2 cleavage and the changes in intracellular and total Nav1.1α expression. It also restored sodium currents density in hippocampal neurons and neuronal activity, as indicated by EEG tracing; improved Morris water maze performance; and shifted APP amyloidogenic metabolism towards non-amyloidogenic processing. There were no differences in these indicators between WT and Navβ2-kd mice. These results suggest Navβ2 knockdown may be a promising strategy for treating AD.

## INTRODUCTION

Alzheimer’s disease (AD) is the most common form of dementia and affects as many as 35 million people worldwide [1]. AD incidence is expected to increase rapidly as the population ages. AD patient cognitive deficits and full-time care needs exact tremendous emotional and financial burden on family members and the health care system. Thus, developing viable anti-AD therapies is an urgent necessity [2]. Although some approved treatments can alleviate AD symptoms, an improved understanding of AD pathogenesis is required to enable development of more effective, potentially curative treatments [3].

Epileptic activity can occur at early AD stages and might contribute to pathogenesis. Such seizures can hasten cognitive decline, highlighting the clinical necessity of early disease recognition and treatment [4]. In a familial AD mouse model, hypersynchronous network activity associated with seizure susceptibility precedes amyloid β (Aβ) plaque pathology and memory impairment [5]. Transgenic mouse models of AD exhibit brain-wide aberrant neuronal and epileptiform activity [6-10]. These aberrant neuronal activity and/or seizure modulate cognitive deficits in amyloid precursor protein (APP) transgenic mice and patients with amnestic mild cognitive impairment [6, 9-12], and may directly contribute to cognitive deficits early in AD progression [13].

Voltage-gated sodium channels (VGSCs) are complex transmembrane glycoproteins responsible for action potentials in excitable cells. In addition to producing the large, transient current responsible for the action potential upstroke, action potential, sodium channels carry smaller currents at subthreshold voltages that contribute to spontaneous action potentials generation [14]. VGSCs consisted of a large α subunit, Nav1 (encoded by *SCNA* gene), and associated auxiliary Nav2 subunits (Nav2.1-2.4, encoded by *SCN1B-SCN4B* gene), which modulates channel activity. Voltage-gated sodium channels beta 2 (Navβ2, encoded by *SCN2B* gene), an associated auxiliary subunits, is expressed in the central nervous system and in cardiac tissue [15]. Navβ2 causes a depolarizing shift in Nav1.1 and Nav1.6 (encoded by *SCN8A*) voltage dependent activation and inactivation [15, 16]. Nav2 upregulation and diffuse distribution along demyelinated axons may result in recovery from conduction block and clinical remission [17, 18]. Navβ2 has also been implicated in the pathogenesis of multiple sclerosis and experimental acute encephalitis (EAE), because it regulates channel cell surface expression in neurons [19], including that of Nav1, which modulates action potential propagation and neuronal activity [20-22]. Our previous study associated increased hippocampal *SCN2B* transcription in SAMP8 mice with learning and memory deficits, but the underlying mechanisms are still not understood [23].

β secretase, also known as β-site amyloid precursor protein cleaving enzyme 1 (BACE1), initiates the production of the toxic Aβ protein that plays a crucial role in early AD pathogenesis. Like APP, Navβ2 is a single transmembrane domain protein that is cleaved by BACE1 to produce a C-terminal fragment (CTF), which is subsequently cleaved by γ-secretase to release an intracellular domain (ICD) [22, 24]. Increased Navβ2 processing and reduced Nav1.1α surface expression in the transgenic APP mouse cortex suggests that both glutamatergic and GABAergic neurons are susceptible to enhanced Navβ2 processing [13].

The link between Navβ2, neuronal activity, and APP processing in AD has not yet been characterized. Given the roles of Navβ2 in Na+ channel cell surface expression and cognitive (learning and memory) regulation, the present study investigated whether Navβ2 expression changes affect neuronal activity, and/or seizure and spatial cognition via Nav1.1α surface expression regulation and APP processing in an AD mice model.

## RESULTS

### Transgenic mouse genotyping

**Figure 1:**
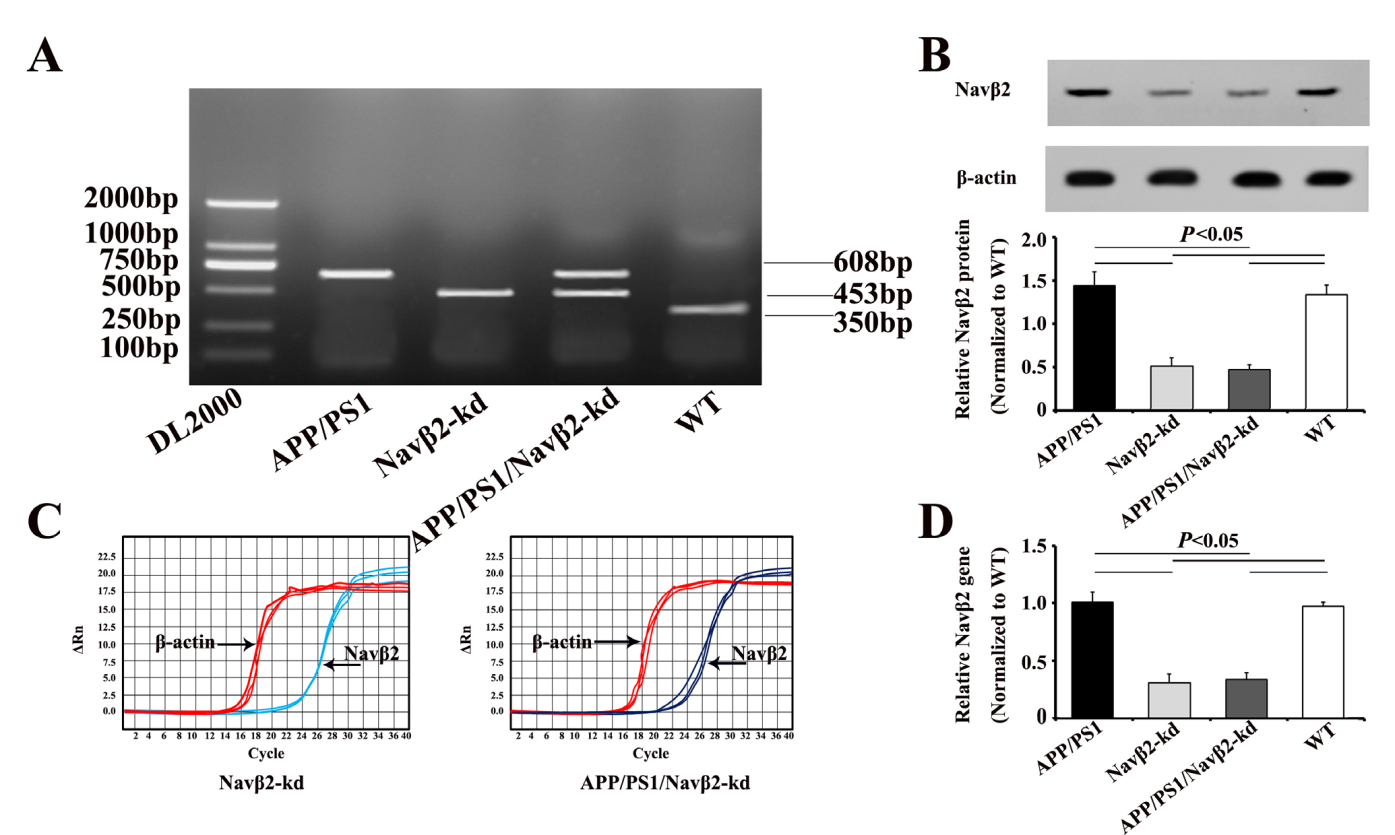
APP/PS1/Navβ2-kd transgenic mouse production PCR products were separated by gel electrophoresis on a 1.5% agarose gel **A**. Navβ2 protein in the Tg mouse hippocampus **B**. Navβ2 expression in the Tg mouse hippocampus as detected by qRT-PCR. qRT-PCR amplification plots **C**. and quantitative expression analysis **D**. Navβ2 expression in Navβ2-kd and APP/PS1/Navβ2-kd mice was reduced by nearly 61%, compared to WT mice. (n=9).

Potential transgenic (Tg) founders were screened for the presence of transgene via PCR using mouse genomic DNA isolated from tail tissue. APP/presenilin 1 (APP/PS1), Navβ2-kd, APP/PS1/Navβ2-kd, and WT mice were screened by PCR (APP/PS1 Tg mice, 608bp; Navβ2-kd mice, 453bp; APP/PS1/Navβ2 mice, 608bp and 453bp; WT mice, 350bp, respectively) (Figure 1A). Navβ2 levels in different transgenic mice were detected via qRT-PCR and western blotting. Navβ2 knockdown reduced Navβ2 protein (Figure 1B) and mRNA levels (Figure 1C-1D) by nearly 61% compared to WT mice (Supplementary Figures 1–2, Supplementary Tables 1–2).

### Navβ2 cleavage and Nav1.1α expression changes in APP/PS1 mice after Navβ2 knockdown

**Figure 2:**
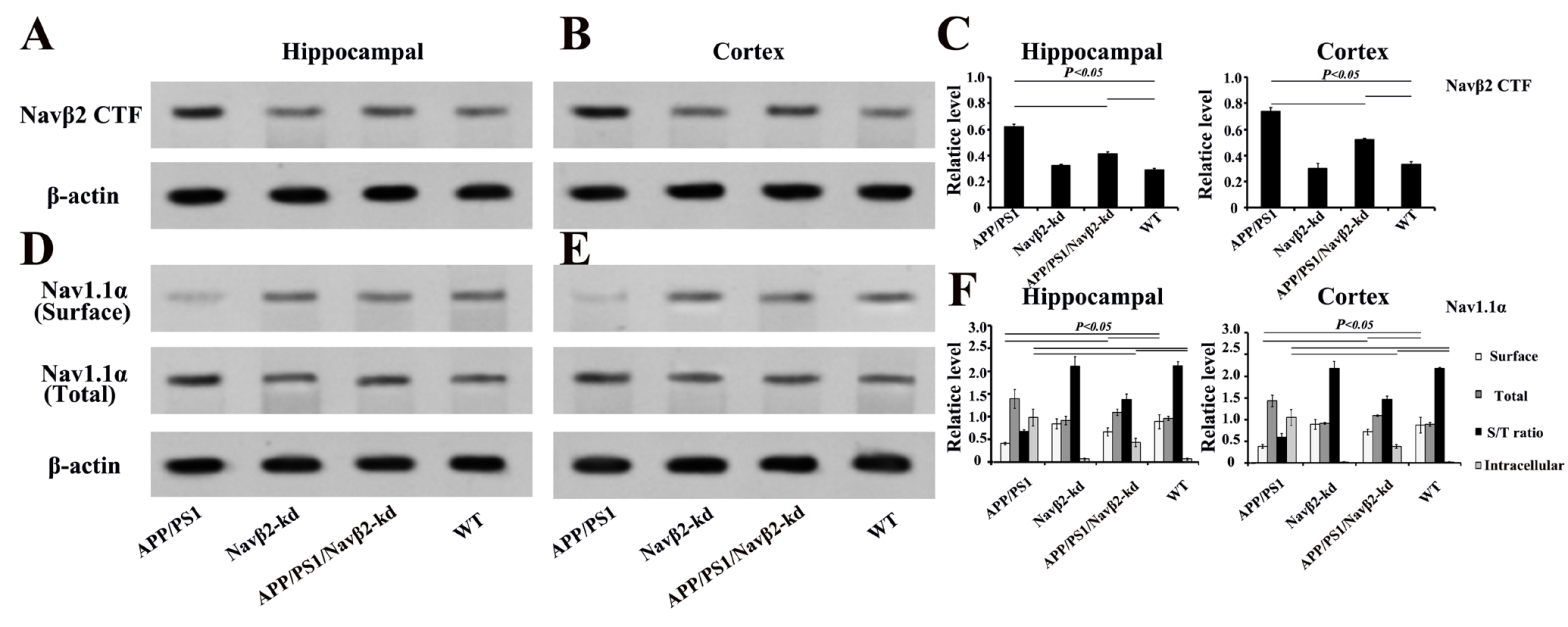
Nav alteration after Navβ2 knockdown in Tg mice Increased Navβ2 CTF in hippocampal **A**. and cerebral cortex **B**. lysates from eight-month-old APP/PS1 compared with APP/PS1/Navβ2-kd, Navβ2-kd or WT mice., Navβ2 CTF expression quantification in different groups **C**. Decreased surface Nav1.1α and increased total Nav1.1α levels in hippocampal **D**. or cortical **E**. tissues of eight-month-old APP/PS1 compared with APP/PS1/Navβ2-kd, Navβ2-kd or WT mice. Surface and total Nav1.1α levels in different groups **F.** (n=15).

Western blot analysis showed Navβ2 knockdown by 61% partially reversed the Navβ2 cleavage and Nav1.1α expression in APP/PS1/Navβ2-kd mice (vs. APP/PS1 mice). Navβ2 CTF (produced by BACE1 cleavage) and total Nav1.1α increased in the hippocampal or cerebral cortex regions of APP/PS1 mice (vs. WT, *P*<0.05, Figure 2A-2C). Navβ2 CTF levels in hippocampal/cortical lysates were lower in APP/PS1/Navβ2-kd mice than in APP/PS1 mice (*P*<0.05). Surface biotinylation assays of hippocampus or cortex slices showed that Nav1.1α was nearly absent on APP/PS1 mouse neuronal cell surface, but was restored in APP/PS1/Navβ2-kd mice (Figure 2D-2F). Intracellular Nav1.1α expression increased in APP/PS1 mice (vs. WT, *P*<0.05), and returned to lower levels following Navβ2 knockdown ( *P*<0.05). Navβ2-kd Tg mice without APP/PS1 mutation exhibited no changes in Navβ2 cleavage and Nav1.1α levels(vs. WT, *P*>0.05, Fig.2). These results suggest that Navβ2 knockdown partially corrected the excessive Navβ2 cleavage and aberrant Nav1.1α transcellular trafficking observed in APP/PS1 mice.

### Navβ2 knockdown recovered sodium currents in APP/PS1 mice

**Figure 3:**
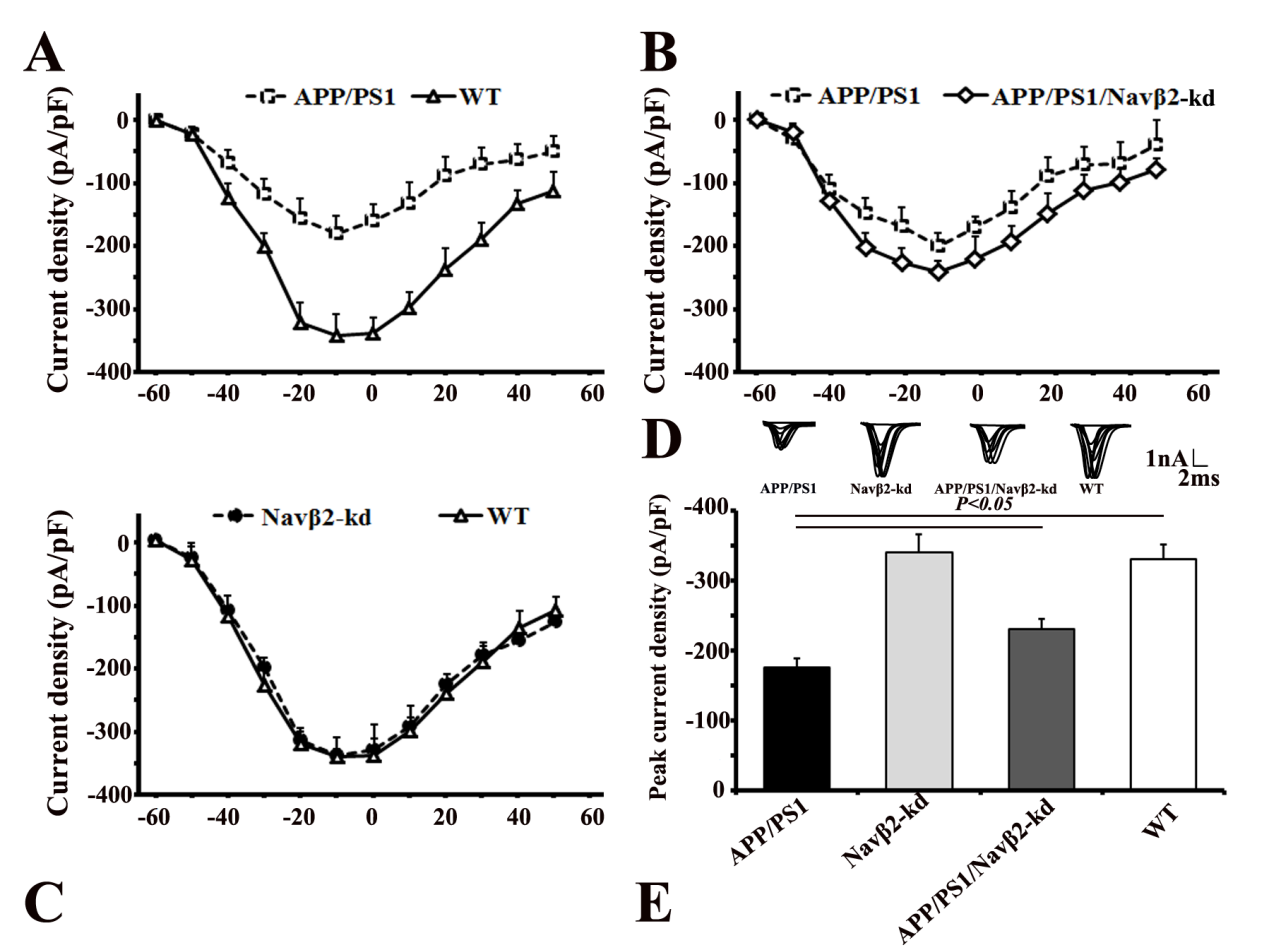
Navβ2 knockdown recovered sodium currents in APP/PS1 Tg mice Whole cell recordings were performed using hippocampal neurons from APP/PS1, Navβ2-kd, APP/PS1/Navβ2-kd, and WT mice. Fast sodium currents were activated by stepped depolarizations, following pharmacological suppression of voltage-dependent calcium and potassium currents with Cd2+ and Cs+, respectively. Peak sodium current amplitude at each voltage step (I-V) was normalized to the neuron’s capacitance and plotted as a function of the command potential (28). Hippocampal slices of APP/PS1, Navβ2-kd, APP/PS1/Navβ2-kd, and WT mice (6-8 weeks) were used for whole cell recordings of voltage-gated sodium currents. Depolarizing voltage steps, from -60 mV to 60 mV (10 mV increments) in neurons from APP/PS1, Navβ2-kd, APP/PS1/Navβ2-kd, and WT mice, were performed to elicit the corresponding sodium current responses. Sodium currents were reduced in hippocampal neurons from APP/PS1 mice. Navβ2 knockdown partially recovered sodium currents in APP/PS1/Navβ2-kd mice (vs. APP/PS1, *P*<0.05). There was no difference between Navβ2-kd and WT mice (*P*>0.05). Current-voltage relationships for sodium current densities in APP/PS1 and WT mice **A** in APP/PS1, APP/PS1/Navβ2-kd, and WT mice **B**., and in Navβ2-kd and WT mice **C**. Representative sodium current responses elicited by depolarizing voltage steps **D**. Peak current densities in mice with different genotypes **E**. (n=15). pF, picofarads.

The peak sodium current densities in hippocampal neurons decreased in APP/PS1 (vs. WT, *P*<0.05, Figure 3A & 3E) and partially recovered in APP/PS1/Navβ2-kd mice (vs. APP/PS1, *P*<0.05), as shown by sodium current I-V curves (Figure 3B) and peak currents densities (Figure 3E). The results suggest that Navβ2 knockdown recovered sodium current densities in APP/PS1 mouse cells. However, Navβ2-kd mouse sodium current densities did not differ from those pf WT mice (*P*>0.05, Figure 3C & 3E).

### Navβ2 knockdown mitigated aberrant neuronal activity in APP/PS1 mice

**Figure 4:**
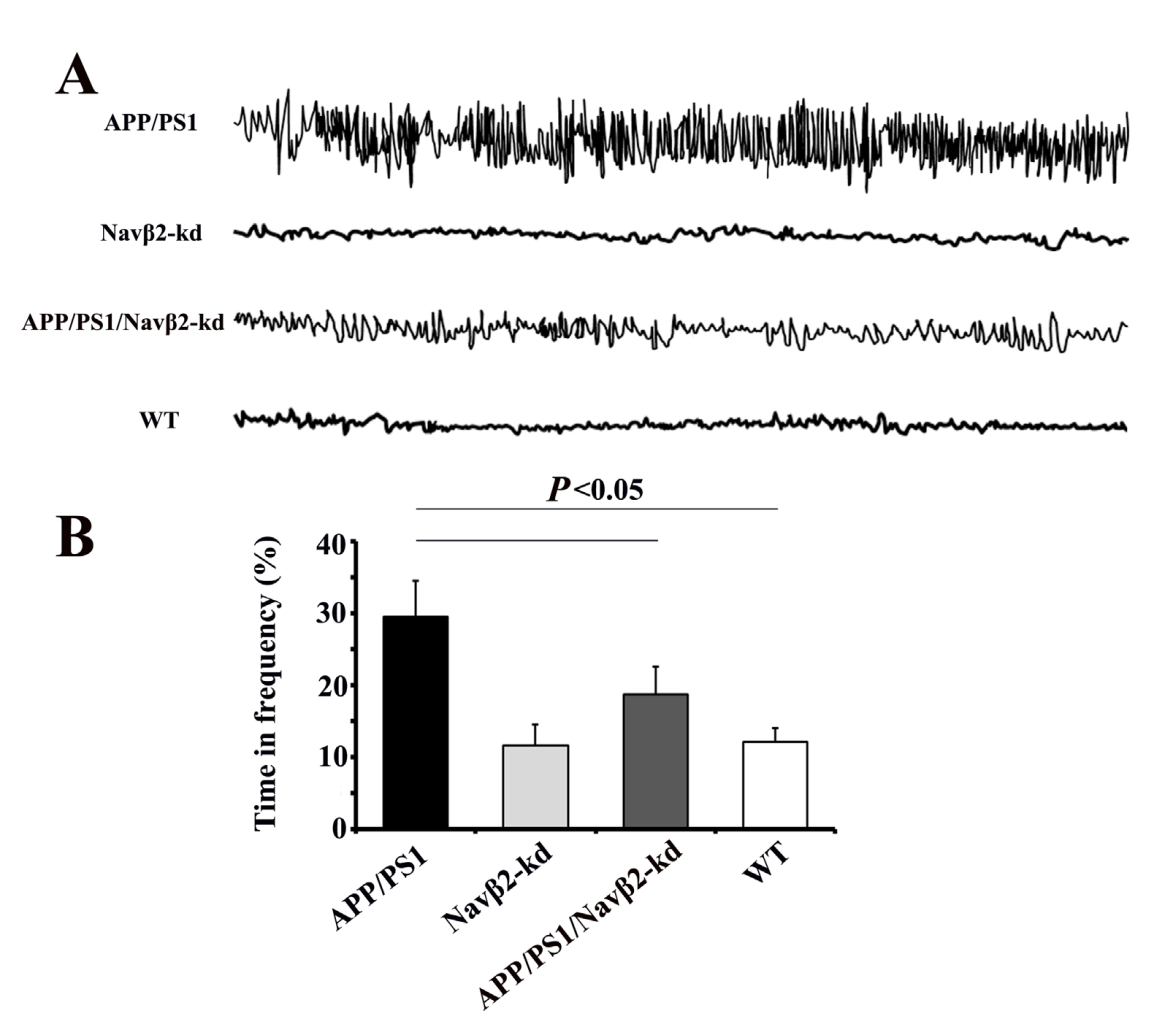
Navβ2 knockdown reversed aberrant neuronal activity in APP/PS1 mice EEG recordings showed increased brain activity amplitudes and frequencies in eight-month-old APP/PS1 mice, and this was partially reversed in APP/PS1/Navβ2-kd mice **A**. Percentage of time at frequency > 6Hz **B**. APP/PS1 mice exhibited longer durations of higher frequency activity than APP/PS1/Navβ2-kd, Navβ2-kd and WT mice (n=15).

We performed video-EEG recordings and MWM test to determine whether Navβ2 knockdown affected neuronal activity. We found that eight-month-old APP/PS1 mice exhibited spike-wave discharges (SWDs) and abnormal EEG patterns compared to WT mice (Figure 4A). These abnormal EEG patterns were at least partially mitigated in Navβ2 knockdown APP/PS1 mice (Figure 4A). APP/PS1 mice exhibited longer durations of higher frequency brain activity with some obvious spikes. APP/PS1/Navβ2-kd mice, with decreased Navβ2 cleavage and total levels of Nav1.1α, exhibited more comparative normal neuronal activity without epileptiform discharges (vs. APP/PS1, *P*<0.05, Figure 4B). However, this recovery did not reach normal levels (APP/PS1/Navβ2-kd vs. WT, *P*<0.05, Figure 4B). There was no difference between Navβ2-kd and WT mice (*P*>0.05).

### Navβ2 knockdown improved learning and memory of APP/PS1 mice

**Figure 5:**
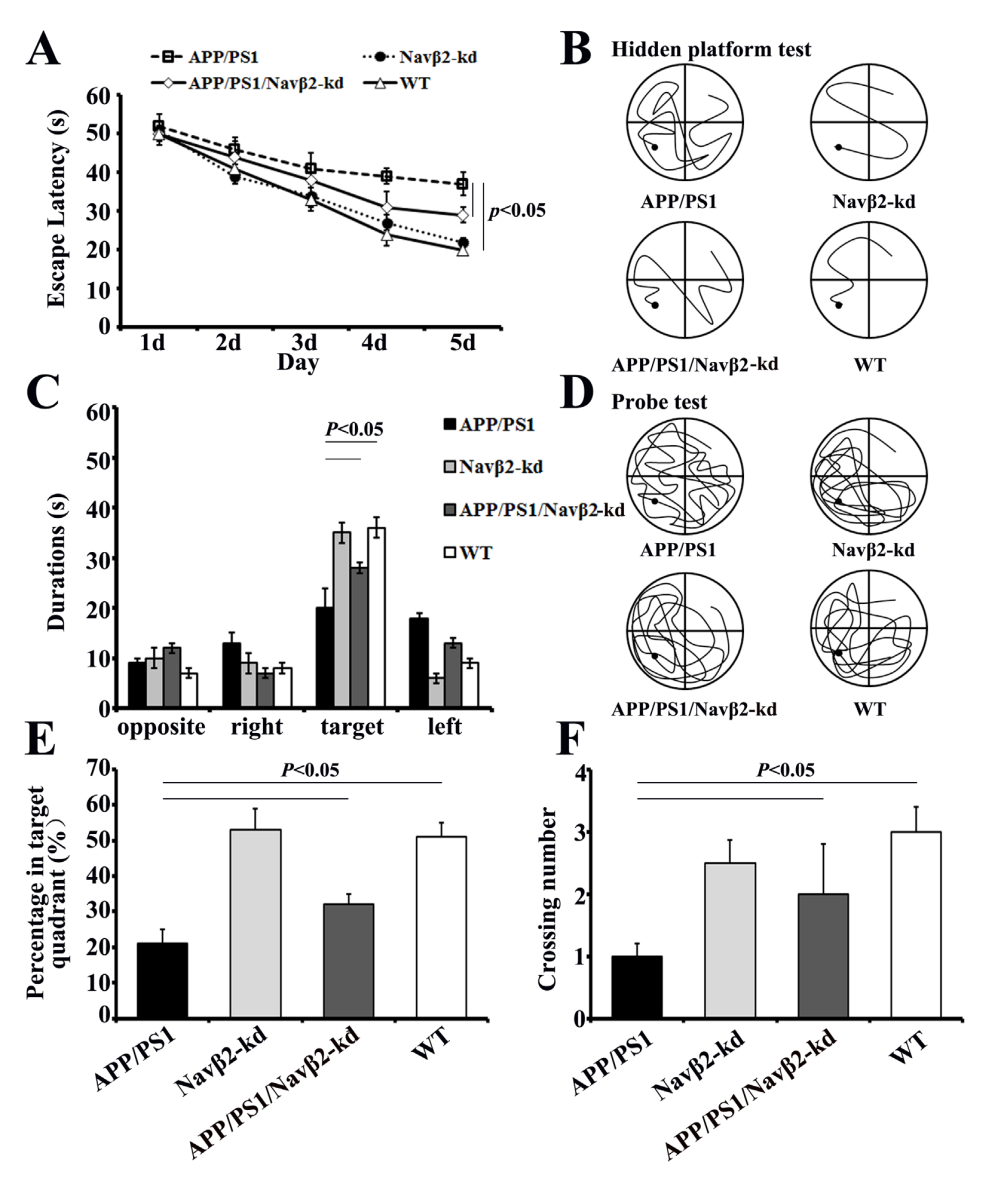
Spatial learning and memory were improved in APP/PS1/Navβ2-kd Tg mice Escape latencies over five days in the hidden platform test **A**. and typical training traces (fifth day) **B**. Comparisons of the percentage of time spent in the target quadrant **C**., percentage of path travelled in the target quadrant **D**., and number of target crossings in the probe test **E**. for APP/PS1, Navβ2-kd, APP/PS1/Navβ2-kd, and WT mice. Typical probe traces **F**. (n=15).

Tow-way repeated-measures ANOVA (RM ANOVA) revealed that the average escape latency of the hidden platform progressively decreased over time in all groups. APP/PS1 Tg mice needed more time to find the hidden platform underwater than both WT (F= 25.28, *P*<0.000) and APP/PS1/Navβ2-kd mice (F= 17.15, *P*<0.000) (Figure 5A). At the end of the hidden platform test, the platform was removed for the probe trial and all mice were allowed to swim for 60 s to assess their memory for the platform’s location. APP/PS1 mice showed lower percentages of time in the target quadrant (tow-way ANOVA Tukey’s Test, *P*<0.05, Figure 5B), percentages of path in the target quadrant (tow-way ANOVA Tukey’s Test, *P*<0.05, Figure 5C) and numbers of platform crossings than age-matched WT (tow-way ANOVA Tukey’s Test, *P*<0.05, Figure 5D).

Navβ2 knockdown in APP/PS1 mice partially improved MWM performance, although spatial memory didn’t recover to WT levels. APP/PS1/Navβ2-kd mice took less time to find the hidden platform (F= 9.41, *P*<0.05, Figure 5A), showed a significant preference for the target quadrant (*P*<0.05, Figure 5C), had lower percentages of path in the target quadrant (*P*<0.05, Figure 5D), and fewer platform crossings (*P*<0.05, Figure 5E) than age matched APP/PS1 mice. Additionally, we observed no spatial learning and memory performance difference in eight-month-old Navβ2-kd mice compared to age matched WT mice (*P*>0.05).

### APP processing and Aβ production changes in APP/PS1/Navβ2-kd mice

**Figure 6:**
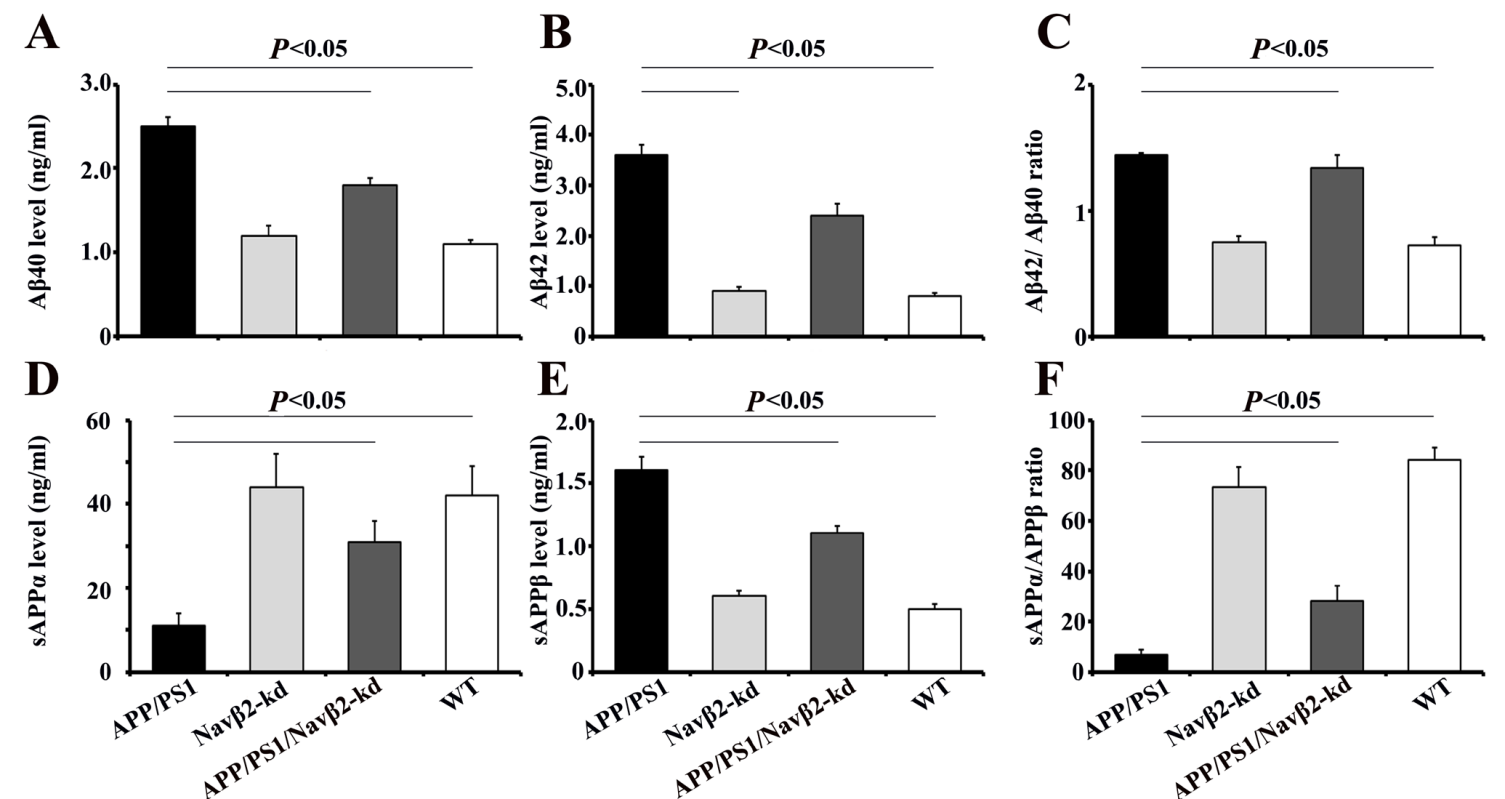
Navβ2 knockdown altered APP processing in an APP/PS1 cell model APP amyloidogenic processing inhibition was confirmed by ELISA measurements of Aβ40 **A**. and Aβ42 **B**. production in culture medium of hippocampus neurons from APP/PS1, Navβ2-kd, APP/PS1/Navβ2-kd, and WT mice. Aβ42/Aβ40 ratio **C**. Results are shown as Aβ levels in ng/ml. Soluble APPα **D**. and sAPPβ **E**. levels in culture medium of cells sourced from mice were monitored via ECLIA. sAPPα/APPβ ratio was calculated as shown in **F**. Results are given as sAPP in ng/ml (n=15).

To determine whether Navβ2 alteration is related to APP metabolism, we measured Aβ40, Aβ42, sAPPα, and sAPPβ levels produced by primary cultured mouse neurons. We detected higher Aβ40 and Aβ42 levels, and an increased Aβ42/Aβ40 ratio in APP/PS1 mouse-derived cells (vs. WT, *P*<0.05, Figure 6A-6C). Decreased Aβ40 and Aβ42 deposition was observed in APP/PS1/Navβ2-kd mice (vs. APP/PS1, *P*<0.05, Figure 6A-6B). There were no differences in Aβ40 and Aβ42 levels between WT and Navβ2-kd mice (*P*>0.05).

ECLIA results showed sAPPα downregulation (vs. WT, *P*<0.001), sAPPβ upregulation (vs. WT, *P*<0.001), and decreased sAPPα/sAPPβ ratios (vs. WT, *P*<0.05) in APP/PS1 mouse-derived cells (Figure 6D-6F). APP/PS1/Navβ2-kd mouse-derived cells showed sAPPα upregulation (vs. APP/PS1, *P*<0.05), sAPPβ downregulation (vs. APP/PS1, *P*<0.001), and an increased sAPPα/sAPPβ ratio (vs. APP/PS1, *P*<0.001). There was no difference between Navβ2-kd and WT mice (*P*>0.05).

## DISCUSSION

Previous studies demonstrated that five- to six-month-old pre-plaque APP mice exhibited Nav1.1α upregulation and increased Navβ2 CTF levels [13]. We examined whether Navβ2 knockdown regulates Navβ2 cleavage and Nav1.1α expression in an AD mouse model. Navβ2 knockdown in APP/PS1 mice reversed abnormal Navβ2 cleavage and both intracellular and total Nav1.1α expression, , recovered sodium currents densities in hippocampal neurons, partially rectified abnormal EEG tracing, improved MWM performance, and shifted APP/PS1 mutation-induced APP amyloidogenic metabolism toward non-amyloidogenic processing. Thus, aberrant neuronal network excitability attributed to cognitive deficits in APP/PS1 mice might be at least partially remedied by Navβ2 knockdown. However, Navβ2 cleavage, Nav1.1α levels, sodium currents densities, and spatial learning and memory were unchanged in Navβ2-kd Tg mice lacking the APP/PS1 genetic background compared to WT mice.

Normal Navβ2 expression and function are necessary for Nav1.1α transcellular trafficking and surface expression [30]. Navβ2 CTF produced by BACE1 is subsequently cleaved by γ-secretase to produce Navβ2-ICD, which translocates to the nucleus and triggers expression of the pore-forming Nav1.1α subunit [22, 24]. Navβ2 knockdown partially restored Navβ2 processing and Nav1.1α expression.

VGSCs are responsible for action potentials in excitable cells. In addition to producing the large, transient current responsible for the action potential upstroke, sodium channels carry smaller currents at subthreshold voltages that contribute to the generation of spontaneous action potentials [14]. Excessive Navβ2 cleavage induced by BACE1 overexpression in a Tg mouse model retained intracellular levels and reduced surface levels of Nav1.1α, leading to decreased action potential propagation and neuronal activity [21, 22]. In agreement with our findings, alterations in cortical Nav1.1α levels were associated with aberrant EEG activity, and correlated inversely with water maze performance [13]. We proposed that Navβ2 knockdown might partially recover sodium current densities and aberrant epileptic seizures incidences in AD model by altering Navβ2 cleavage and retaining surface Nav1.1α levels.

Epileptic seizures are common in AD patients, and hyper-excitability is detected in AD transgenic mouse brains [31]. Such aberrant increases in network excitability and compensatory inhibitory mechanisms in the hippocampus may contribute to AD-associated cognitive deficits [6, 11, 32]. EEG indicates the average electrical activity of the neuronal populations on a large-scale level, and are widely utilized as a noninvasive brain monitoring tool in cognitive neuroscience and as a diagnostic tool for epilepsy and sleep disorders [29]. Coordinated cortical activity and oscillations strongly influence function of the hippocampus, which receives spatial information from different cortical areas [33, 34]. Spatial information encoding depends on integration of information from numerous dynamically interconnected regions [35, 36].

Deposition of Aβ, derived from APP by sequential proteolytic cleavage, forms the neuritic plaques that are the pathological hallmark of AD. APP cleavaged by α-secretase precludes Aβ generation as part of the non-amyloidogenic pathway. Conversely, APP processed via amyloidogenic pathway generates a membrane-anchored C-terminal fragment, which is further cleaved by the γ-secretase complex to generate the Aβ peptides. The Aβ isoforms, Aβ40 and Aβ42, are the major constituents of AD-associated senile plaques [37]. Our data detected higher Aβ40, Aβ42, and sAPPβ levels, and lower sAPPα and sAPPα/sAPPβ ratio in APP/PS1 mice. Navβ2 knockdown in these mice decreased Aβ40 and Aβ42 deposition and sAPPβ production, as well as increased sAPPα levels and sAPPα/sAPPβ ratios. Ectodomain shedding indicated by soluble APPα (sAPPα) and sAPPβ production is the first step in non-amyloidogenic and amyloidogenic processing, respectively [38]. Navβ2 knockdown shifted APP/PS1 mutation-induced APP amyloidogenic metabolism towards non-amyloidogenic processing.. Li, *et al.* reported that APP/AICD modulates Nav1.6 sodium channels through a Go-coupled JNK pathway, which is dependent on APP phosphorylation at Thr668 [28]. Others revealed that *Scn2b*−/− mice are protected from axonal damage during EAE due Nav1.6 downregulation along demyelinated axons, thus reducing the harmful effects of the predicted persistent Na+ current. The absence of β2 may also attenuate the persistent Na+ current mediated by Na+ channels in demyelinated lesions [19]. Moreover, treatment with Aβ increased Nav and Nav1.6 expression in cultured neurons, indicating that Aβ1-42 could alter the number of Nav channels [39]. Thus, our findings suggested that Navβ2 expression might indirectly or directly modulate APP metabolism by interacting with Nav1.6, although further investigation was necessary.

Our previous study demonstrated that a downregulation of *SCN2B* (by 60.68%) improved learning and memory, and increased hippocampal synaptic excitability in aged (18-month-old) mice, and did not impact adult (eight-month-old) mice [21]. Our present work found no differences in Navβ2 cleavage, surface Nav1.1α levels, sodium currents densities, neuronal activity, or spatial learning and memory between eight-month-old Navβ2-kd and age-matched WT mice.. O’Malley, *et al.* demonstrated that the regulation of Na+ channel cell surface expression and function within special microenvironments, such as neuronal injured or demyelination, is critical to neuronal survival and recovery [19]. Therefore, we assumed that Navβ2 downregulation protected neurons by a cleavage condition-depended mechanism. Normally, Navβ2, as an auxiliary subunit, assists Nav1 subunit in maintaining sodium gate function. Navβ2 pathological cleavage triggered during early stage AD reduces surface Nav1.1α levels, induces aberrant neuronal activity, amyloidogenic processing, and ultimately cognitive deficit.

In conclusion, we demonstrated that Navβ2 knockdown protected neurons and improved spatial learning and memory by partially reversing the excessive Navβ2 cleavage, aberrant Nav1.1α transcellular trafficking, reduced sodium channel densities and neuronal activity, and APP amyloidogenic metabolism observed in aged APP/PS1 mice. Our findings suggest that Navβ2 knockdown might be a promise anti-AD therapeutic strategy.

## MATERIALS AND METHODS

### Ethics approval

Animal use and care were in conducted in accordance with guidelines provided in the Guide for the Care and Use of Laboratory Animals published by the US National Institutes of Health (NIH Publication No. 85-23, revised 1996) and in the Care and Use Guidelines of Experimental Animals established by the Research Ethics Committee of Kunming University of China (Permit Number: kmu-eac-2016019). All surgical procedures were performed under diethyl ether anaesthesia, and all efforts were made to minimize suffering.

### Animal model generation

APPswe/PS1ΔE9 (APP/PS1, C57BL/6J) mice, in which APP overexpress with the Swedish mutation and PS1 is deleted in exon 9, rapidly accumulates Aβ plaques at eight months of age. Navβ2 knockdown (Navβ2-kd) transgenic (Tg) mice with C57BL/6J genetic background were generated and bred in our lab as previously described [23]. Our previous study associated increased hippocampal Navβ2 in aged SAMP8 mice with learning and memory deficits. A 60.68% *SCN2B* transcription reduction improved hippocampus-dependent spatial recognition memory and field excitatory postsynaptic potential (fEPSP) long-term potentiation (LTP) slope in aged mice [23]. The present study investigated the neuroprotective effects of Navβ2 knockdown in APP/PS1 AD mouse model.

APP/PS1, APP/PS1/Navβ2-kd mice with C57BL/6J genetic background were generated and bred by our collaborators in the Institute of Laboratory Animal Science (Chinese Academy of Medical Sciences & Comparative Medicine Centre, Peking Union Medical College, Beijing, China). The *SCN2B* silencing vector and reconstruction plasmid were designed as previously described [23]. The transgene was then isolated from the cloning plasmid, purified using Avr II digestion, and microinjected into fertilized APP/PS1 mouse eggs.

Overexpression of human APP with the Swedish (K594M/N595L) mutation, deletion of PS1 in exon 9 driven by the mouse prion protein promoter, and Navβ2 knockdown were confirmed by PCR genotyping of APP/PS1/Navβ2-kd Tg mouse tail tissue [23, 25] (APP/PS1 Tg mice, 608bp; Navβ2-kd mice, 453bp; APP/PS1/Navβ2 mice, 608bp and 453bp; WT mice, 350bp). Potential Tg founders were screened for the presence of the transgene via PCR using mouse genomic DNA isolated from tail biopsies. The following primers were used for Navβ2-kd mice: sense, 5’GCTCGGTGTTGCTGTGAT 3’; antisense, 5’TGATGGGCTACGGCTTCT 3’. The following primers were used for APP/PS1 mice:: transgenic forward, 5’AAT AGA GAA CGG CAG GAG CA 3’, transgenic reverse, 5’GCC ATG AGG GCA CTA ATC 3’; Internal control forward, 5’CTA GGC CAC AGA ATT GAA AGA TCT 3’, Internal control reverse, 5’ GTA GGT GGA AAT TCT AGC ATC ATC C 3’. RT-PCR products were electrophoresed in 1.2% agarose gel, stained with ethidium bromide (EB), and visualized using an ultra violet gel imager (BIO-GEL, BIO-RAD). Image analysis was performed by Imager J 6.0 (LIVE Science, USA).

Since the female APP/PS1 mice develop cognitive deficits faster than their male counterparts, only female mice were used in this study [26]. Because APP/PS1 induced greater cognitive impairment in aged mice, experiments were performed using APP/PS1/Navβ2-kd triple Tg mice aged eight months. Age-matched APP/PS1, Navβ2-kd and wild type (WT) mice were used as controls.

### Western blot

Hippocampus and cortex from the mice with different genotypes (n=15 per group) were harvested and separately homogenized on ice in 10 mM Tris-HCl buffer (pH 7.4), 10 mM EDTA, 30 % Triton-1000, 10 % SDS, protease inhibitors cocktails (Roche), and NaCl, using a homogenizer. Homogenates were centrifuged at 5,000g for 10 min at 4 °C. Equal amounts of protein (quantized by BCA method) were resolved by SDS-PAGE on 4-12% gels, transferred to nitrocellulose, and incubated with antibodies against either Navβ2 (1:500, Alomone) or Nav1.1α (1:800, Alomone). β-actin (mouse monoclonal anti-β-actin, 1:800, Santa Cruz, Delaware, CA) was used as a reference. Nitrocellulose membranes were washed and incubated with peroxidase-conjugated anti-mouse secondary antibodies (1:10,000, Santa Cruz), and were detected using enhanced chemiluminescence reagent (Pierce). Signals were quantified using a ChemiDoc™ XRS+ imaging system with Image Lab™ software (Bio-Rad, USA).

### Quantitative RT-PCR

Samples were obtained for Navβ2 detection via reverse transcription and quantitative real-time PCR (qRT-PCR) as previously described [27]. The relative CT method was used to compare difference between samples. Fold decrease/increase was determined relative to a blank control after normalized to a housekeeping gene β-actin housekeeping gene using 2^-ΔΔCT^.

### Cell surface biotinylation assay

Hippocampal and cortical cortex tissues were collected from Tg mice (eight months old) with different genotypes (n=15 in each group). Cell surface biotinylation and detection of surface Nav1.1α subunits were performed as previously described [13, 23]. NeutrAvidin-agarose beads (Pierce) were employed to pull down biotinylated proteins.

### Morris Water Maze Test

The Morris water maze (MWM) consisted of a circular pool (100 cm diameter, 50 cm deep) filled with water at 24-26°C to a depth of 20 cm. The MWM test followed as previously described [23]. Percentages of time spent in the target quadrant, path in the target quadrant, and number of target platform crossings were recorded.

### Electrophysiological patch clamp recording

Brains were removed from anesthetized 7-8 weeks-old APP/PS1, Navβ2-kd, APP/PS1/Navβ2-kd, and WT mice (n=15 per group), and placed in anoxygenated (95% O_2_/5% CO_2_) artificial cerebrospinal fluid (ACSF) at 4 °C. Whole cell recordings of voltage-gated sodium currents in hippocampal slices from different genotype mice were performed as previously described [28]. Cells were clamped using a MultiClamp 700B amplifier (Molecular Devices Corp., Sunnyvale, CA, USA) in conjunction with a Digidata 1322 A interface (Axon Instruments, Union City, CA, USA) at a holding potential of -70 mV. pCLAMP 9.2 software (Molecular Devices Corp.) was used for current recording and analyzing. Voltage command protocols were as described previously [28]. The peak amplitude of the second sodium current response divided by the response at the maximal interval was plotted as a function of the interpulse interval. Curves were fitted with a double rising exponential function.

### Electroencephalogram (EEG) recordings

APP/PS1, Navβ2-kd, APP/PS1/Navβ2-kd (eight months old) and age matched WT littermate mice were anesthetized for EEG recordings by using nanofabricated polyimide-based microelectrodes (PBM-array) (n=15 per group). PBM-array surgical implantation on mouse skulls and EEG recordings were performed as previously described [29] with some modifications.

EEG signals were interpolated to 128 Hz and bandpass filtered from 1 to 64 Hz. Power spectral densities (PSDs) were generated every 30 s for each recording to calculate the amount of time spent at >6 Hz (time in high frequency). Signal processing ensured that integer values represented the dominant frequency (DF) in Hz for each 30 s epoch. Average DF was calculated for each mouse from each DF in each 30 s epoch. Time in high frequency was calculated by summing the number of 30 s epochs with a DF >6 Hz and dividing it by the total number of 30 s epochs (total time of the recording) for each mouse [13].

### Cell culture

The hippocampus of APP/PS1, Navβ2-kd, APP/PS1/Navβ2-kd, and WT mice were completely removed following craniotomy. These were dissected into 1mm^3^ slices in PBS using an anatomic microscope, and then digested for 30 min at 37°C using 2mg/ml Papain (Roche) containing 2μl/ml DNAase. Digestion was terminated by addition of an equal volume of Dulbecco’s modified Eagle’s medium (DMEM) supplemented with 10% fetal bovine serum (FBS) and 1% penicillin-streptomycin. The cell suspension was centrifuged at 1,000 rpm for 10 min at 4°C. After discarding the supernatant, cells were re-suspended in the same medium by gently pipetting up and down and were seeded on 24-well plates at 2×10^5^ cells/ml. Medium was replaced with Neurobasal (GIBCO) medium supplemented with 2% B27 supplement and 1% penicillin/streptomycin.

### ELISA detection of Aβ levels

After five days of culture, mediums was collected from APP/PS1, Navβ2-kd, APP/PS1/Navβ2-kd, and WT mouse cell cultures. Aβ40 and Aβ42 levels were measured via ELISA (Demeditec Diagnostics GmbH, German) according to the manufacturer’s instructions. Aβ levels in cell culture media were compared between different genotypes.

### Solution APP measurement

A sAPPα/sAPPβ multiplex electrochemiluminescence assay (ECLIA; Meso Scale Discovery) kit was used to quantify sAPPα and β. Before harvesting, cells were conditioned in serum-free medium for 16 h. Cell medium was then collected and sAPPα and β were quantified according to the manufacturer’s instructions.

### Statistical analysis

We used the SPSS 19.0 for Windows covariance software package for statistical analysis. Results are expressed as the means ± standard deviation (SD). Differences between two groups were evaluated using Student’s t test. One-variable experiments with more than two groups were evaluated using ANOVA followed by Bonferroni’s post hoc tests. Electrophysiological patch clamp recording data were analyzed via one-way ANOVA, followed by Student’s t-test for paired groups (two tailed). Tow-way repeated-measures ANOVA followed by Tukey’s test were employed for MWM test analysis. *P*<0.05 was considered significant.

## SUPPLEMENTARY MATERIALS FIGURES AND TABLES


