# Supplementary material for: Navβ2 knockdown improves cognition in APP/PS1 mice by partially inhibiting seizures and APP amyloid processing
Source: Oncotarget. 2017 Oct 16;8(59):99284–95. doi: 10.18632/oncotarget.21849 (PMC5725092; doi:10.18632/oncotarget.21849)
Supplement: Supplementary file 1 [file oncotarget-08-99284-s001.pdf]

Navβ2 knockdown improves cognition in APP/PS1 mice by partially inhibiting seizures and APP amyloid processing

SUPPLEMENTARY MATERIALS

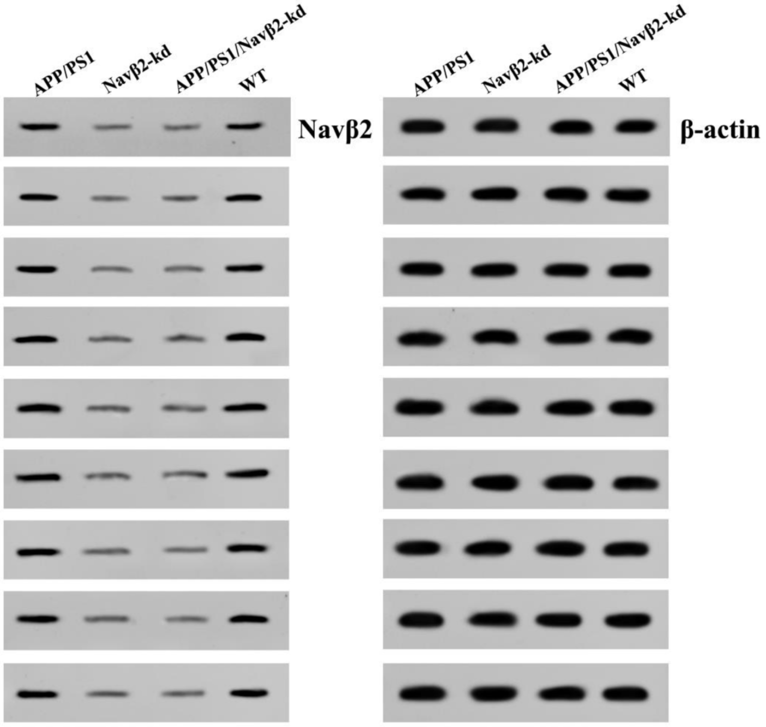

Supplementary Figure 1: Expression of Navβ2 protein in hippocampus of different transgenic mice by Western blot. The original blots of Navβ2 protein in hippocampus from different groups detected by Western blot (n=9).

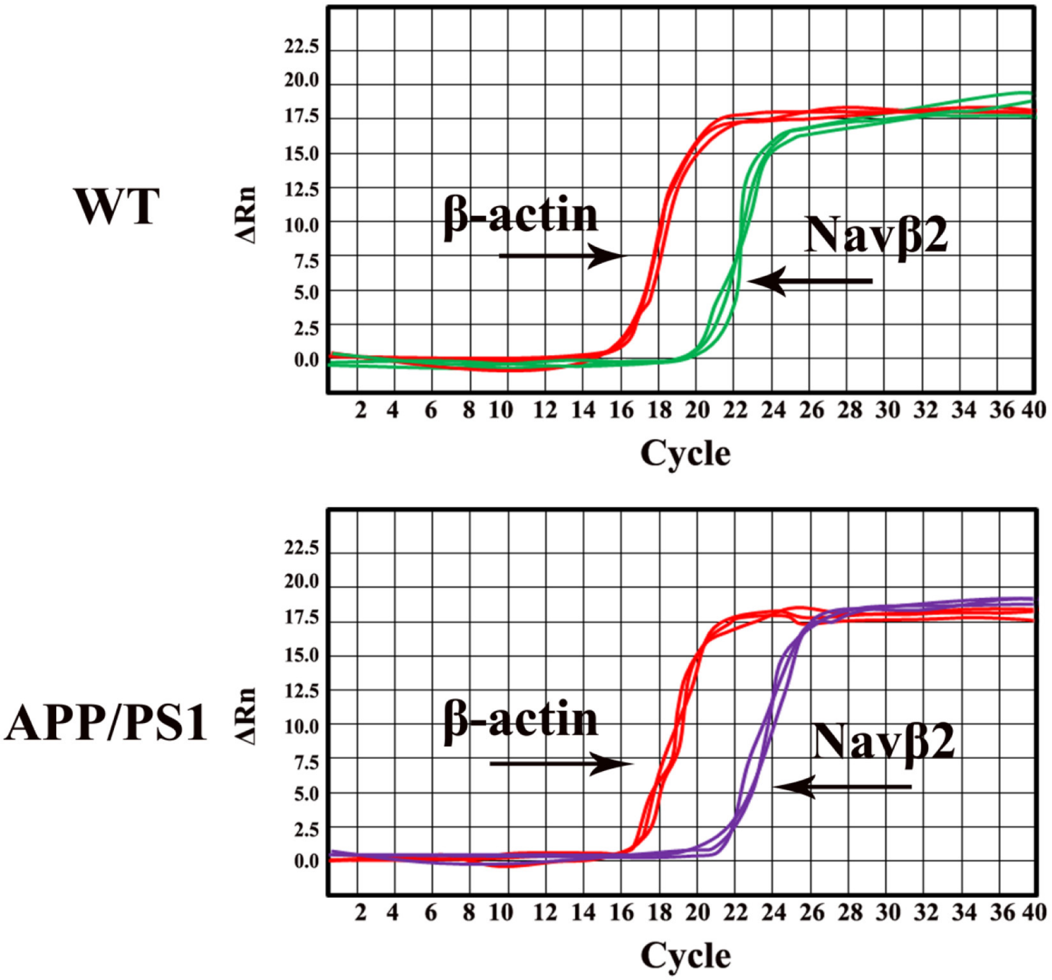

Supplementary Figure 2: Amplification plots of qRT-PCR for Navβ2 gene expressions in APP/PS1 and WT group.

Supplementary Table 1: Quantitative analysis of Navβ2 protein in hippocampus of mice from different groups (n=9)

Group	Relative Navβ2 protein levels	
	Navβ2/β-actin	Normalized to WT
**APP/PS1**	0.49 ± 0.09*^	1.04 ± 0.12*^
**Navβ2-kd**	0.17 ± 0.04#&	0.39 ± 0.07#&
**APP/PS1/ Navβ2-kd**	0.15 ± 0.03#&	0.36 ± 0.04#&
**WT**	0.46 ± 0.05*^	1.0 ± 0.09*^

Data are presented as means ± SD (n=9)
* vs APP/PS1/ Navβ2-kd, *P*<0.05;
# vs APP/PS1, *P*<0.05;
& vs WT, *P*<0.05;
^ vs Navβ2-kd, *P*<0.05

Supplementary Table 2: Navβ2 gene expression in hippocampus of different transgenic mice detected by qRT-PCR

Group	Relative Navβ2 protein levels (Normalized to WT)
**APP/PS1**	1.04± 0.09*^
**Navβ2-kd**	0.37± 0.08#&
**APP/PS1/ Navβ2-kd**	0.39± 0.06#&
**WT**	1.0 ± 0.04*^

Data are presented as means ± SD (n=9)
* vs APP/PS1/Navβ2-kd, *P*<0.05;
# vs APP/PS1, *P*<0.05;
& vs WT, *P*<0.05;
^ vs Navβ2-kd, *P*<0.05
